# The mRNA decay factor tristetraprolin (TTP) induces senescence in human papillomavirus-transformed cervical cancer cells by targeting E6-AP ubiquitin ligase

**DOI:** 10.18632/aging.100086

**Published:** 2009-09-10

**Authors:** Sandhya Sanduja, Vimala Kaza, Dan A. Dixon

**Affiliations:** Department of Biological Sciences and Cancer Research Center, University of South Carolina, Columbia,; SC 29203, USA

**Keywords:** tristetraprolin, senescence, AU-rich element, HPV, E6-AP

## Abstract

The RNA-binding protein tristetraprolin (TTP) regulates expression of many cancer-associated and proinflammatory factors through binding AU-rich elements (ARE) in the 3'-untranslated region (3'UTR) and facilitating rapid mRNA decay. Here we report on the ability of TTP to act in an anti-proliferative capacity in HPV18-positive HeLa cells by inducing senescence. HeLa cells maintain a dormant p53 pathway and elevated telomerase activity resulting from HPV-mediated transformation, whereas TTP expression counteracted this effect by stabilizing p53 protein and inhibiting hTERT expression. Presence of TTP did not alter E6 and E7 viral mRNA levels indicating that these are not TTP targets. It was found that TTP promoted rapid mRNA decay of the cellular ubiquitin ligase E6-associated protein (E6-AP). RNA-binding studies demonstrated TTP and E6-AP mRNA interaction and deletion of the E6-AP mRNA ARE-containing 3'UTR imparts resistance to TTP-mediated downregulation. Similar results were obtained with high-risk HPV16-positive cells that employ the E6-AP pathway to control p53 and hTERT levels. Furthermore, loss of TTP expression was consistently observed in cervical cancer tissue compared to normal tissue. These findings demonstrate the ability of TTP to act as a tumor suppressor by inhibiting the E6-AP pathway and indicate TTP loss to be a critical event during HPV-mediated carcinogenesis.

## Introduction

Cervical cancer is the second most common cancer among women worldwide [1]. A necessary factor in the development of nearly all cases of cervical cancer is infection with the high-risk human papillomavirus (HPV) types 16 and 18 [2]. These subtypes of HPV promote cellular transformation through expression of the early viral genes *E6 *and *E7*. The HPV E7 protein neutralizes the retinoblastoma (Rb) tumor suppressor pathway by sequestering Rb from E2F and promoting its destabilization [3,4], while the E6 protein promotes degradation of the p53 tumor suppressor and activates transcription of the human telomerase reverse transcriptase gene (*hTERT*) [5,6].  The oncogenic functions of E6 occur through its interaction with a number of cellular regulatory proteins and one of the best characterized E6-binding partners is the E6-associated protein (E6-AP) [7,8]. E6-AP belongs to a class of HECT ubiquitin-protein ligases [9] and its interaction with E6 facilitates cell transformation through enhanced p53 protein degradation and activation of *hTERT* gene expression [10]. Deregulation of these critical factors through the combined action of E6 and E7 oncoproteins allows for continued cell proliferation and genomic instability ultimately leading to HPV-mediated cellular transformation.

Messenger RNA turnover is a tightly regulated process that plays a central role in controlling mammalian gene expression. The significance of this is evident in disease and tumorigenesis where loss of post-transcriptional gene regulation accounts for the aberrant overexpression of a variety of genes encoding growth factors, inflammatory cytokines and proto-oncogenes [11,12]. A majority of cancer-associated immediate-early response genes that control growth and inflammation display conserved *cis*-acting adenylate- and uridylate (AU)-rich elements (ARE) in the mRNA 3' untranslated region (3'UTR). A primary function of the ARE is to target mRNAs for rapid decay through interaction with *trans-*acting RNA-binding proteins that have high affinity for AREs. Among the best characterized ARE-binding proteins involved in promoting ARE-mediated mRNA decay is tristetraprolin (TTP, ZFP36, TIS11). TTP is a member of a small family of tandem Cys3His zinc finger proteins originally identified as an inducible immediate-early response gene [13]. Initially thought to be a transcription factor, various studies have established the role of TTP as an mRNA decay protein that binds to AREs in the mRNA of various inflammatory mediators (e.g. TNF-α, GM-CSF, COX-2) [14-16]. The binding of TTP to ARE-mRNAs targets them for rapid degradation through association with various decay enzymes [14,17-21]. The physiological role of TTP is significant as TTP deficient mice develop a number of inflammatory syndromes. These abnormalities have been shown to be due to excessive levels of pro-inflammatory factors resulting from defects in ARE-mediated decay in these mice [22,23].

In this study, we examined the role of TTP in HPV-mediated cervical carcinogenesis. Expression of TTP in HPV18-positive HeLa cells dramatically inhibited cell growth by inducing cellular senescence through a mechanism involving p53 protein stabilization and inhibition of telomerase expression. It was found that TTP induced cellular senescence through rapid decay of E6-AP ubiquitin ligase mRNA that was mediated through the ARE-containing 3'UTR of E6-AP. Furthermore, we demonstrate that TTP expression is lost in cervical cancer compared to normal tissue, implying a tumor suppressor function for TTP in cervical tissue. These novel findings not only add another attribute to the already established anti-inflammatory role of this ARE-binding protein but also bring new insights into the mechanism of HPV-mediated cervical carcinogenesis.

## Results

### TTP-mediated induction of senescence in HeLa cells

**Figure 1.**
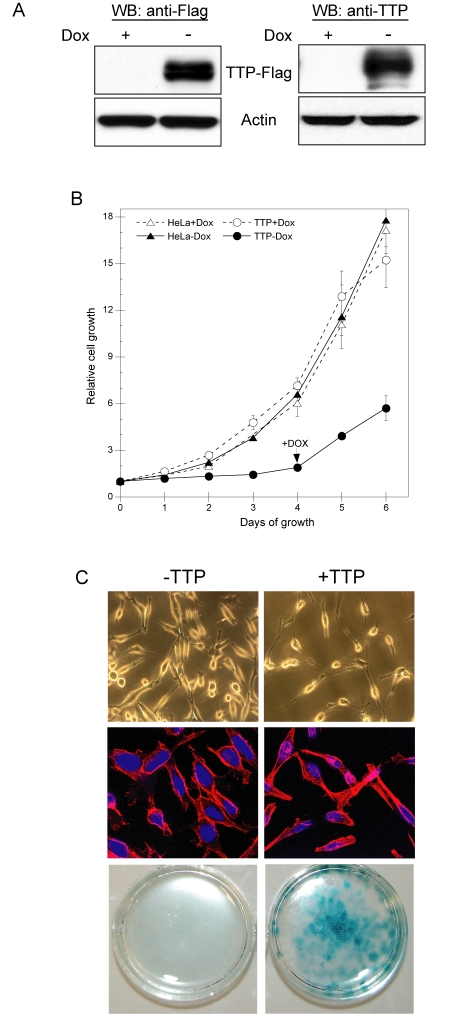
TTP inhibits HeLa cell proliferation through induction of senescence. (**A**) HeLa Tet-Off/TTP-Flag cells grown in the presence or absence of 2 μg/ml Dox for 48 hr. The expression of TTP-Flag was detected by western blot (WB) using antibodies against the Flag epitope (left panel) or TTP (right panel). Actin was used as a loading control. (**B**) Growth curves of HeLa Tet-Off/TTP-Flag (circles) and parental HeLa Tet-Off (triangles) cells in the presence (open symbols) or absence (filled symbols) of 2 μg/ml Dox. On day 4 of growth, Dox was added to HeLa Tet-Off/TTP-Flag cells to repress TTP expression. Each point represents the mean of 4 replicates. (**C**) HeLa Tet-Off/TTP-Flag cells were grown in the presence or absence of Dox to repress (- TTP) or induce (+ TTP) TTP, respectively. Phase contrast (top panels) and fluorescence (middle panels) microscopy of cells after 48 hr of TTP expression; original magnification 200X and 400X, respectively. Nuclei (blue) and cytoskeleton (red) are shown in fluorescent micrographs. HeLa-Tet-Off/TTP-Flag cells were stained for SA-β-gal activity (bottom panels) after 12 days of TTP expression.

Based on its ability to control expression of ARE-containing mRNAs associated with various aspects of cellular transformation and tumorigenesis, TTP can serve  in a tumor suppressor capacity. To test this in HPV-transformed cervical carcinoma cells, a tetracycline (Tet)-regulated TTP expression system in HeLa cells was developed. HeLa Tet-Off cells were stably transfected with a Flag epitope-tagged TTP cDNA in a Tet-regulated expression vector such that cells grown in the absence of doxycycline (Dox) allow for the expression of TTP (Figure 1A). Consistent with other findings [24,25], endogenous TTP expression was undetectable in HeLa cells and in HeLa Tet-Off parental cells grown in the presence or absence of Dox (Figure 1A and data not shown).

To determine the consequence of TTP expression, we evaluated the ability of TTP to attenuate HeLa cell growth and proliferation. As shown in Figure 1B, HeLa Tet-Off/TTP-Flag cells grown in the absence of Dox showed a marked reduction in proliferation and this effect was dependent on TTP; re-addition of Dox to turn-off TTP expression allowed for increased cell growth. Consistent with this, a decreased rate of DNA synthesis was observed in HeLa cells expressing TTP and similar results were obtained from 3 other independent HeLa Tet-Off/TTP-Flag clones (data not shown). Interestingly, HeLa cells grown in the presence of TTP for 48 hr exhibited a flattened morphology resembling cells that had undergone replicative senescence (Figure 1C, [26]). Upon longer exposure to TTP (12 days), these cells contained elevated levels of senescence-associated β-galactosidase (SA β-gal) [27] further indicating the ability of TTP to attenuate HeLa cell growth through a mechanism involving cellular senescence.

### TTP promotes p53 expression through protein stabilization

**Figure 2.**
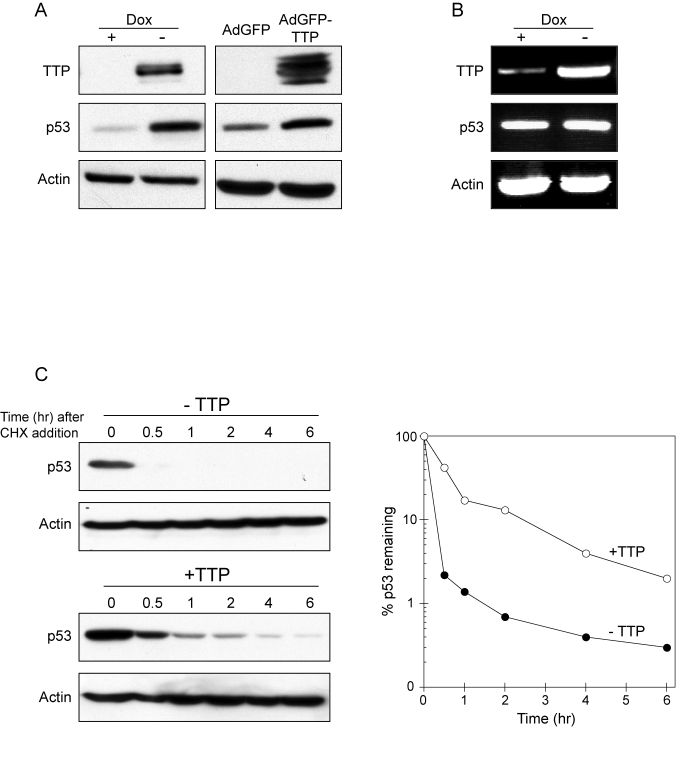
TTP promotes p53 expression through protein stabilization. (**A**) HeLa Tet-Off/TTP-Flag cells grown in presence or absence of Dox for 48 hr (left panel) and HeLa cells infected with AdGFP or AdGFP/TTP virus for 48 hr (right panel) were examined for TTP and p53 expression by western blotting. Actin was used as a loading control. (**B**) RT-PCR analysis of p53 mRNA expression in HeLa Tet-Off/TTP-Flag cells grown in presence or absence of Dox for 48 hr. Induction TTP-Flag mRNA is shown along with loading control GAPDH. (**C**) TTP promotes increased stability of p53 protein. HeLa Tet-Off/TTP-Flag cells grown in presence (- TTP) or absence (+ TTP) of Dox for 48 hr were incubated with 20 μg/ml cycloheximide (CHX) to inhibit protein synthesis for the indicated times. Decay of p53 protein was examined by western blot (left panels) using actin as a loading control. Decay curves of p53 protein (right panel) in the presence (open circles) and absence (filled circles) of TTP was obtained by western blot analysis and normalized to the internal control actin.

HPV oncogenicity is mediated through the interaction between HPV E6 protein and the tumor suppressor p53 with E6 promoting accelerated ubiquitin-mediated degradation of p53 [7,10]. Based on this, we sought to determine if the growth inhibitory effect exerted by TTP was being modulated through p53 activation. As shown in Figure 2A, induction of TTP in HeLa Tet-Off/TTP-Flag cells resulted in increased expression of p53 protein. Similarly, infection of HeLa cells with an adenovirus expressing TTP resulted in enhanced p53 protein expression as compared to cells infected with control adenovirus expressing GFP (Figure 2A). The ability of TTP to promote p53 expression appeared to be through protein stabilization since p53 mRNA levels were not respectively increased with TTP induction (Figure 2B). To specifically test this, HeLa Tet-Off/TTP-Flag cells were grown in presence or absence of TTP for 48 hr and then treated with cycloheximide (CHX) to inhibit protein synthesis. In the presence of TTP, the half-life of p53 protein was increased 3-fold (Figure 2C), indicating the ability of TTP to inhibit p53 protein turnover.

**Figure 3.**
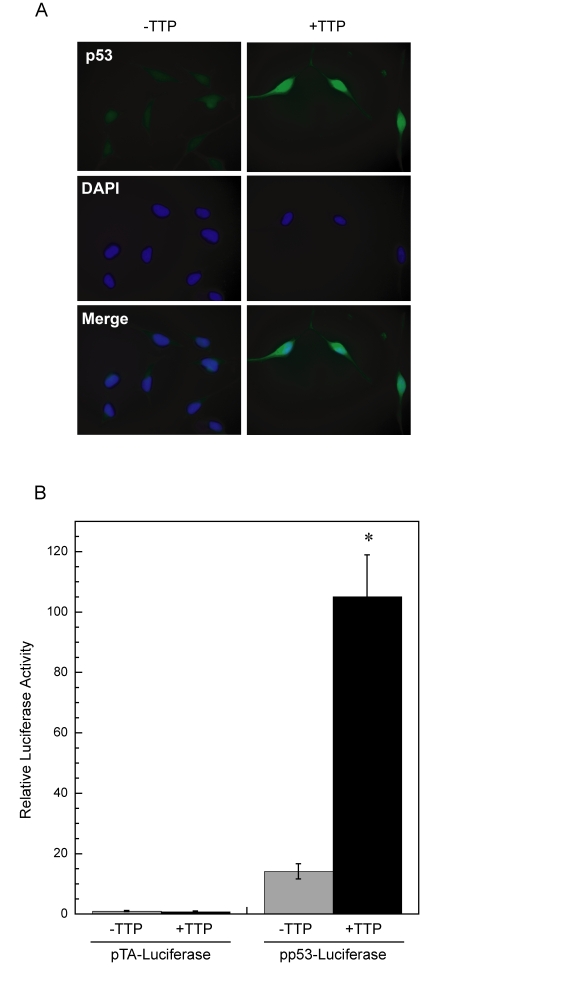
Enhanced p53 activity in HeLa cells expressing TTP. (**A**) Immunofluorescent detection of p53, shown in green, in HeLa Tet-Off/TTP-Flag cellsin the absence or presence of TTP for 48 hr. DAPI nuclear staining and merged images are shown. (**B**) Expression of TTP induces p53 transcriptional activity. Luciferase reporter constructs containing either a p53-dependent promoter (pp53-Luciferase) or control vector (pTA-Luciferase) were transfected into HeLa Tet-Off/TTP-Flag cells and allowed to grow without (grey bars) or with (black bars) TTP induction for 48 hr. Relative activity was assessed as luciferase activity normalized to renilla activity and are the averages of 3 experiments. (*) *P* < 0.01

Activation of p53 promotes its accumulation in the nucleus and transcription of p53-responsive promoters [28,29]. To determine if nuclear localization of p53 is occurring in cells expressing TTP, HeLa Tet-Off/TTP-Flag cells were examined for p53 localization by immunofluorescence. In cells grown in the presence of TTP, p53 was detected in both the nucleus and cytoplasm with a high level of p53 localized to the nucleus (Figure 3A). In parallel experiments, a reporter construct containing a p53-dependent promoter was transfected into HeLa Tet-Off/TTP-Flag cells and its activity was examined in the presence and absence of TTP (Figure 3B). The magnitude of promoter activity was significantly increased in the presence of TTP, consistent with observed p53 protein stabilization and nuclear localization promoted by TTP.

### TTP expression downregulates telomerase activity

**Figure 4.**
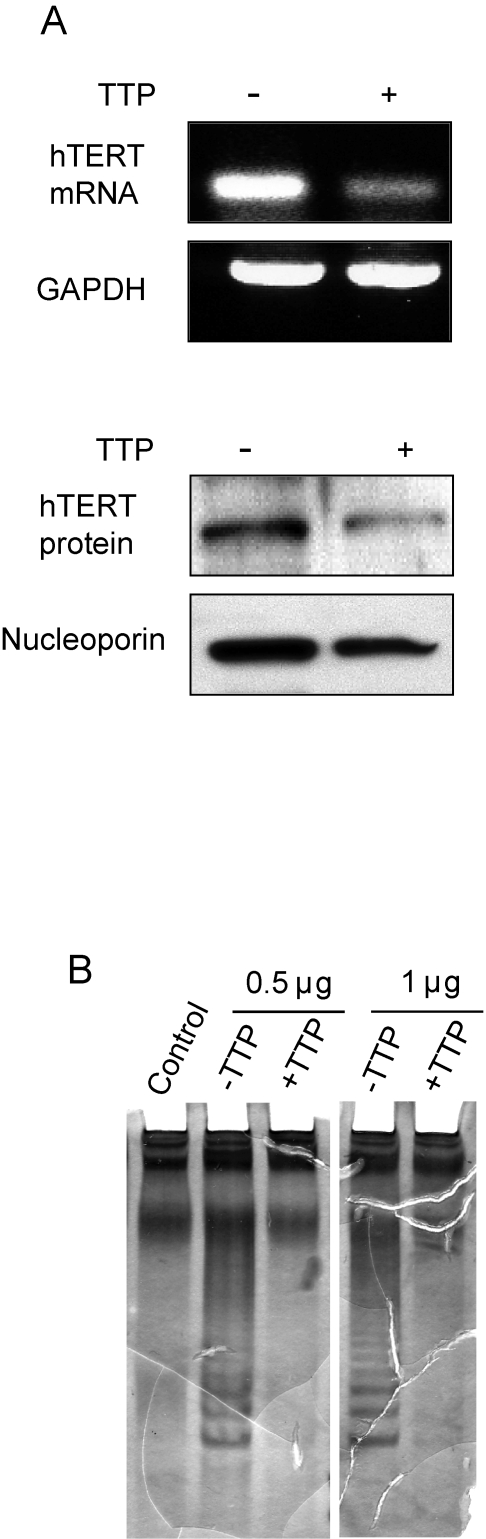
TTP-mediated inhibition of hTERT expression. (**A**) hTERT expression in HeLa Tet-Off/TTP-Flag cells growing in absence or presence of TTP for 48 hr was examined by RT-PCR analysis (top panel) and western blot using nuclear lysates (bottom panel). GAPDH and nucleoporin were detected as loading controls, respectively. (**B**) TRAP assay showing inhibition of telomerase activity in TTP-expressing cells. 0.5 and 1 μg of lysate from cells grown in the absence or presence of TTP was used for TRAP assay as described in Methods. Control reaction lacks Taq polymerase.

Elevated telomerase activity is associated with approximately 85% of human cancers [30]. In cervical cancers, the HPV E6 protein induces telomerase activity by promoting expression of the catalytic subunit of telomerase, hTERT [31]. To determine if TTP expression impacted hTERT levels, HeLa Tet-Off/TTP-Flag cells were grown in the presence and absence of Dox for 48 hr and hTERT mRNA and protein was evaluated. As shown in Figure 4A, steady state levels of both hTERT mRNA and protein were dramatically reduced in presence of TTP. Consistent with this inhibition, a decrease in telomerase activity was also detected (Figure 4B). In cells expressing TTP, a decrease in the characteristic laddering using a telomeric repeat amplification protocol (TRAP) assay was observed indicating that TTP inhibits telomerase activity through inhibition of hTERT expression.

### TTP promotes downregulation of E6-AP ubiquitin ligase

**Figure 5.**
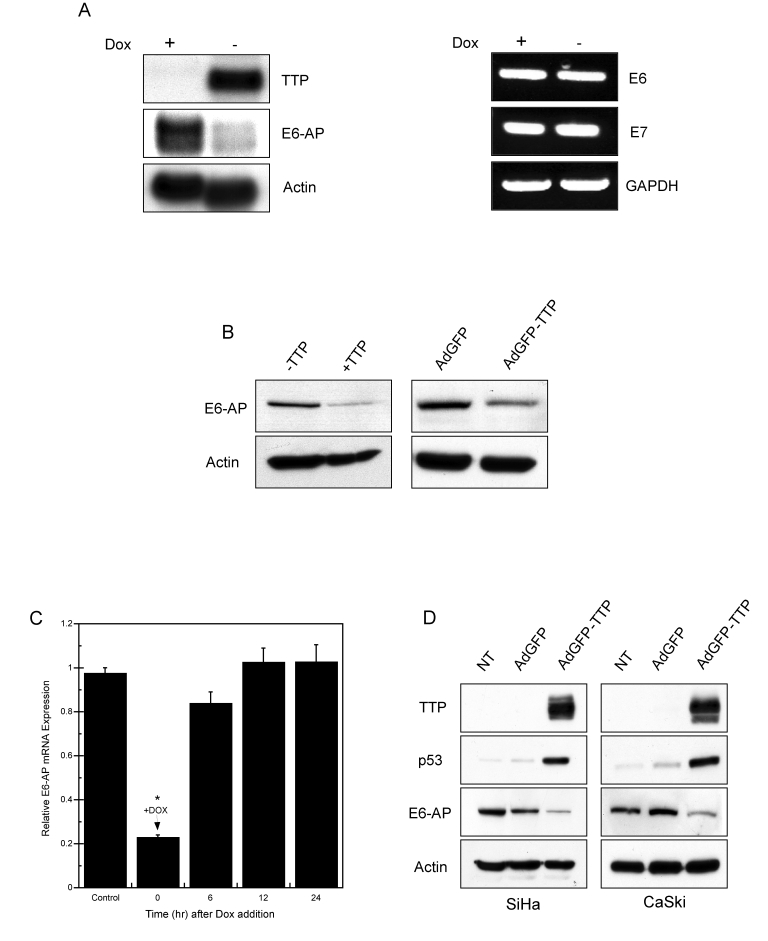
TTP downregulates E6-AP mRNA and protein expression. (**A**) Northern blot (left panel) of TTP and E6-AP mRNA in HeLa Tet-Off/TTP-Flag cells 48 hr after TTP induction. RT-PCR assay (right panel) of HPV18-E6 and -E7 RNA levels in TTP-expressing cells. Actin and GAPDH were used as loading controls. (**B**) Western blot of E6-AP protein in HeLa Tet-Off/TTP-Flag cells (left panel) and adenovirus-infected HeLa cells (right panel) expressing TTP for 48 hr. Actin was used as a loading control. (**C**) TTP-dependent downregulation of E6-AP mRNA. HeLa Tet-Off/TTP-Flag cells were initially grown without Dox for 48 hr to induce TTP. At time zero, Dox was added to the culture medium and E6-AP mRNA levels were evaluated by qPCR over the indicated time course. E6-AP mRNA levels were normalized to control GAPDH mRNA. Cells grown in presence of Dox were used as control. All values shown are normalized to E6-AP expression of control-treated cells and are the averages of 3 experiments. (*) *P* < 0.01 (**D**) HPV 16-positive cells, SiHa (left panel) and CaSki (right panel) were infected with control AdGFP or AdGFP/TTP virus at an MOI of 100 or left untreated (NT). 48 hr after infection, cell lysates were examined for TTP, p53, and E6-AP expression by western blot. Actin was used as a loading control.

In high-risk HPV-transformed cells, the cellular ubiquitin ligase E6-associated protein (E6-AP) plays a central role in mediating the oncogenic functions of E6. E6-AP couples with E6 to target p53 for proteasomal degradation [32]. This complex also degrades the 91 kDa isoform of NFX1 (NFX1-91) which is a repressor for the hTERT promoter, allowing for constitutive hTERT expression in HPV-positive cells [5,33]. Based on this, we examined if TTP could inhibit E6-AP expression in order to establish a molecular explanation underlying TTP's ability to promote senescence. As shown in Figure 5AB, HeLa Tet-Off/TTP-Flag cells grown in the presence of TTP showed downregulation of both E6-AP mRNA and protein. Similarly, HeLa cells infected with adenovirus expressing TTP also showed inhibition of E6-AP expression (Figure 5B, right panel). As a control, the RNA levels of HPV18-E6 and -E7 were assayed (Figure 5A, right panel) and no change was observed in the presence of TTP, indicating that the viral transcript is not a target of TTP. Furthermore, re-addition of Dox to TTP-expressing HeLa Tet-Off/TTP-Flag cells to suppress TTP expression allowed for rapid recovery of E6-AP expression (Figure 5C).

### TTP-mediated stabilization of p53 *via *E6-AP downregulation occurs in high-risk HPV positive cell lines

HPV16 and HPV18 high-risk types are most frequently associated with cervical carcinomas [2]. To determine if these results extended to HPV16-positive cervical cancer cells, SiHa (HPV16+) and CaSki (HPV16+) cells were infected with adenovirus expressing TTP or control GFP. As shown in Figure 5D, endogenous TTP was not detected in either SiHa and CaSki cells, whereas adenoviral delivery of TTP led to E6-AP downregulation and elevated levels of p53 protein in both HPV16+ cell lines to a similar extent to that observed in HPV18+ HeLa cells.

### E6-AP is a novel target for TTP-mediated mRNA decay

**Figure 6.**
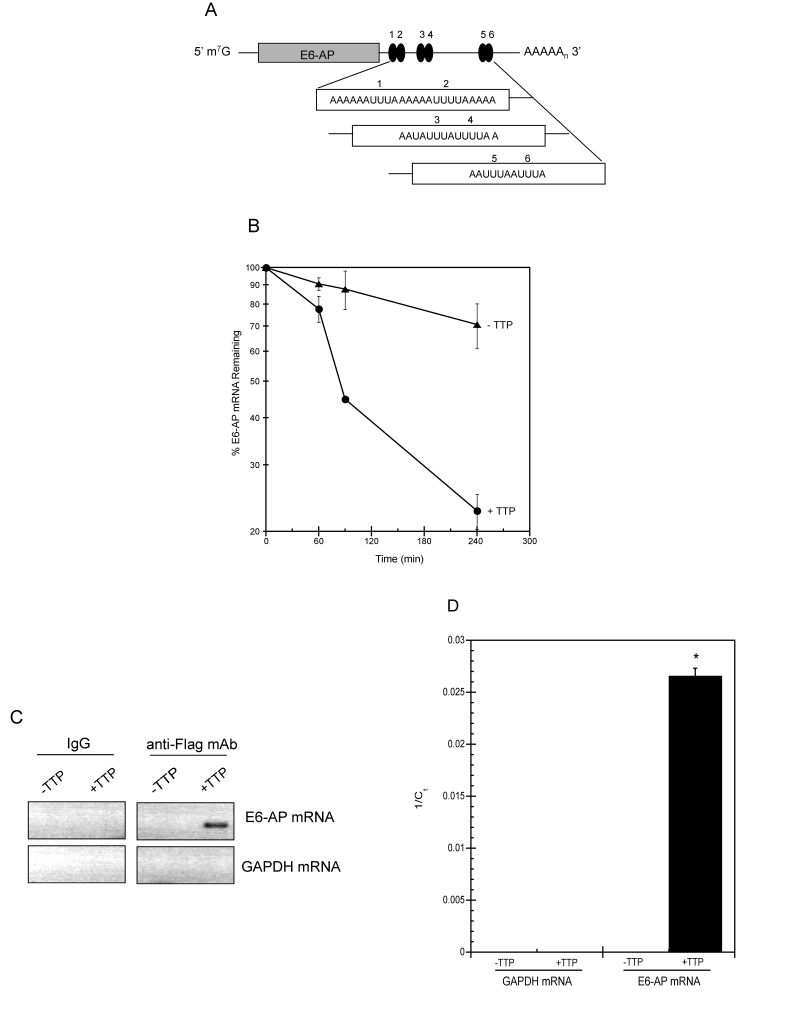
TTP binds E6-AP mRNA and targets it for rapid decay. (**A**) Schematic representation of E6-AP mRNA. The grey bar corresponds to E6-AP coding region and number-labeled black ovals represent putative 3' UTR AU-rich elements (AREs). m7G, 7-methyl-guanosine cap; AAAAn, polyadenylated tail. (**B**) E6-AP mRNA half-life was assayed in HeLa Tet-Off/TTP-Flag cells grown in the presence (triangles; labeled -TTP) or absence (circles; labeled +TTP) of Dox to induce TTP expression. After 48 hr, 5 μg/ml of actinomycin D was added to the cells and E6-AP mRNA decay was analyzed by qPCR using GAPDH mRNA as a normalization control. The data shown is the average of triplicate experiments. (**C, D**) Binding of TTP and E6-AP mRNA. Control and TTP-expressing (48 hr) HeLa Tet-Off/TTP-Flag cells were lysed and immunoprecipitation was performed on equal amounts of cytoplasmic lysates using control IgG or anti-Flag mAb. RNA purified from immuno-precipitates was subjected to RT-PCR (**C**) or qPCR (**D**) to detect E6-AP and GAPDH mRNA. The ethidium bromide-stained agarose gel depicting the 292bp E6-AP PCR product is shown in reverse image. The relative amounts of immuno-precipitated E6-AP mRNA is reported as the average 1/Ct value of triplicate experiments. (*) P < 0.01

Rapid mRNA decay mediated by TTP occurs through *cis*-acting AU-rich RNA elements (AREs) present in the 3'UTR of target transcripts [22,34]. Within the 3'UTR of E6-AP, we detected multiple overlapping copies of AUUUA motif characteristic of Class II AREs (Figure 6A, [35]) suggesting E6-AP mRNA to be a target of ARE-mediated decay. To evaluate this, the half-life of E6-AP mRNA was assessed by qPCR after actinomycin-D (ActD) was added to HeLa Tet-Off/TTP-Flag cells to halt transcription. In cells expressing TTP rapid E6-AP mRNA decay was observed yielding a half-life of approximately 90 min (Figure 6B). In contrast, E6-AP mRNA was stable in cells without TTP with an estimated half-life of 460 min.

To determine if this shortening of half-life was a result of TTP binding to E6-AP mRNA, cytoplasmic extracts from HeLa Tet-Off/TTP-Flag cells grown in the presence and absence of TTP were subjected to immunoprecipitation using anti-Flag antibody or control IgG. Co-immunoprecipitated mRNA was reverse transcribed and PCR amplified using primers specific for E6-AP and GAPDH. Shown in Figure 6C, E6-AP was amplified from TTP expressing cells while no products were detected in control reactions. Samples were also analyzed by qPCR and the *C_t_* values were used to detect the presence of a specific mRNA (Figure 6D). *C_t _*value for E6-AP was 38 in the mRNA pool from TTP expressing cells and undetectable in cells absent of TTP. *C_t_* values were undetectable for GAPDH mRNA indicating its absence in both experimental and control immunoprecipitations.

**Figure 7.**
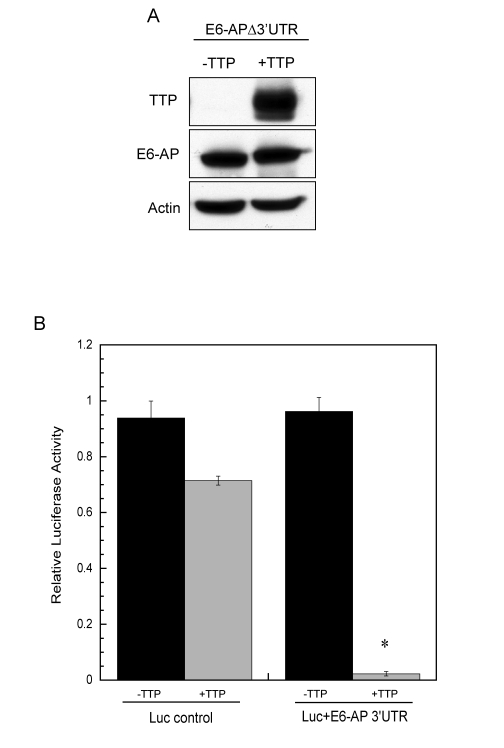
E6-AP 3' UTR is necessary for TTP-mediated decay. (**A**) HeLa Tet-Off/TTP-Flag cells were transfected with an expression vector containing the coding region of E6-AP (E6-APΔ3'UTR). Cells were grown in the absence or presence of TTP for 48 hr and lysates were analyzed for E6-AP and TTP protein expression by western blot. Actin was detected as a loading control. (**B**) HeLa cells were transfected with a luciferase-reporter construct containing the 1.6 kb E6-AP 3'UTR (Luc+E6-AP 3'UTR) or the control luciferase vector (no 3'UTR) along with a TTP expression construct (pcDNA3-TTP-Flag) or empty vector. Relative luciferase reporter activity in the absence (black bars) or presence (grey bars) of TTP is shown. Relative activity was assessed as luciferase activity normalized to its respective protein concentration for each transfection in the absence or presence of TTP. The data shown is the average of duplicate experiments. (*) *P* < 0.01

To determine if the ARE-containing 3'UTR of E6-AP mediated post-transcriptional regulation through TTP,  HeLa Tet-Off/TTP-Flag cells were transiently transfected with a cDNA expression construct containing the 2.5 kb coding region of E6-AP (E6-APΔ3'UTR), and protein expression was assayed in the presence or absence of TTP. We found no TTP-dependent changes in the amount of E6-AP protein expressed when the 3'UTR was absent (Figure 7A) and expression of E6-APΔ3'UTR completely abrogated TTP-mediated stabilization of p53 and subsequent p53-dependent transcriptional activity (data not shown). The ability of the E6-AP 3'UTR to confer TTP-dependent mRNA instability to a reporter was tested by transfecting HeLa cells with a luciferase reporter containing the 1.6 kb E6-AP 3'UTR (Luc+E6-AP 3'UTR) along with a TTP expression construct. As seen in Figure 7B, the E6-AP 3'UTR significantly inhibited luciferase expression in presence of TTP, whereas control transfections using luciferase without a 3'UTR was inhibited by TTP to a much lesser extent. Taken together, these results indicate E6-AP mRNA to be a novel target of TTP-mediated mRNA decay through its ARE-containing 3'UTR.

### TTP expression is lost in cervical cancer

**Figure 8.**
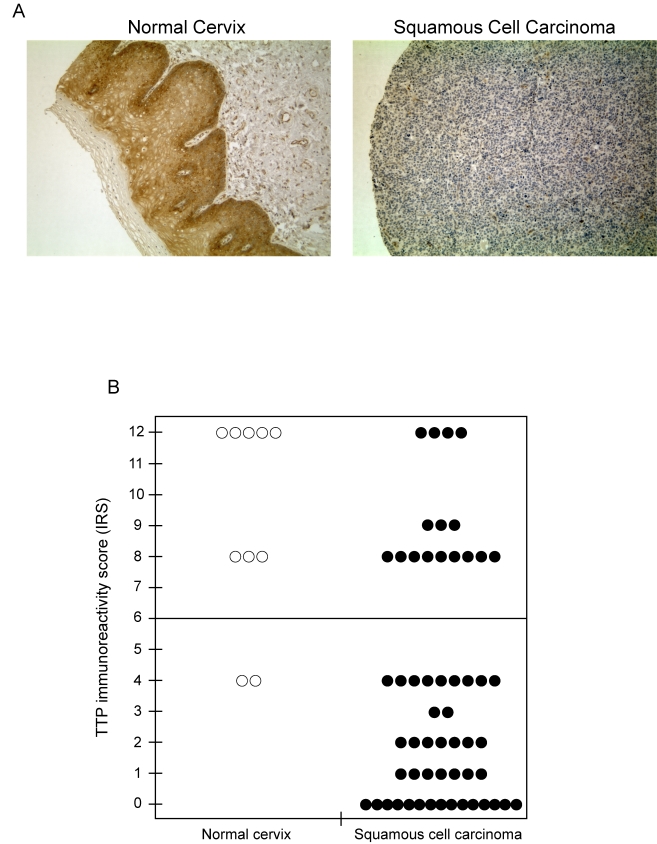
TTP protein expression is lost in human cervical cancer. (**A**) Immunohistochemical detection of TTP expression in normal cervix and squamous cell carcinoma. Representative tissue sections were examined for TTP expression and counterstained with hematoxylin. Original magnification x 200. (**B**) Immunoreactivity scores (IRS) for TTP expression in tissue sections of normal cervix and squamous cell carcinoma. The line indicates the division of samples with high IRS from 7-12 and low IRS from 0-6.

Based on its ability to target E6-AP mRNA for rapid decay, these results suggested that the presence of TTP would be inhibitory to HPV-mediated tumorigenesis and loss of TTP would be observed in cervical cancer. To test this, TTP expression was evaluated by immunohistochemistry using human tissue arrays containing cervical tissue sections from both normal and squamous cell carcinoma (Figure 8A). In normal cervical tissue (left panel) strong cytoplasmic staining of TTP was observed in the cells of squamous epithelium, whereas TTP immunoreactivity was negative or substantially decreased in tissue sections from squamous cell carcinomas (right panel). Tissue sections were assigned immunoreactivity scores (IRS) and grouped as low IRS of 0 to 6 or high IRS of 7 to 12 (Figure 8B). In normal tissue, TTP immuno-reactivity was high (median IRS of 10) in 8 of 10 (80%) samples, while expression was significantly lower (median IRS of 2) in 40 of 56 squamous cell carcinoma samples (71%, *P* < 0.001). These results are consistent with recent findings demonstrating elevated TTP mRNA levels to be present in normal cervix tissue [36] and suggest that loss of TTP expression in cervical cancer cells allows for aberrant mRNA stabilization and enhanced expression of E6-AP.

## Discussion

Normal cellular growth is associated with rapid decay of ARE-containing mRNAs and targeted mRNA decay is an essential way of controlling their pathogenic overexpression. However, a number of observations have implicated loss of ARE-mediated post-transcriptional regulation in the neoplastic transformation of cells [11]. Based upon the inherent genetic instability of tumor cells, it might be expected that mutations in AREs are a frequent event. However, few naturally occurring mutations in AREs have been described [37]. This implies that loss of ARE function in tumor cells is primarily due to altered recognition of AU-rich sequences by *trans*-acting RNA-binding regulatory factors.

Through their ability to selectively bind and control expression of many cancer-associated transcripts [12], ARE RNA-binding proteins are being acknowledged as central regulators influencing various aspects of tumorigenesis. Along with its recognized ability to target rapid decay of an array of inflammatory mediators, the ARE-binding protein TTP has been shown to inhibit expression of a wide range of cancer-associated factors [25,38-44]. Consistent with this, expression of TTP was shown to inhibit cell growth and tumorigenesis in a mast cell tumor model [45] and attenuate colon cancer cell growth and proliferation [44]. These aspects, taken together with the results presented here, indicate that TTP can serve in a tumor suppressor capacity by controlling ARE-containing gene expression.

Through its ability to promote rapid mRNA decay, the tumor suppressor ability of TTP should reflect the ARE-containing mRNAs needed for enhanced tumor cell growth and survival. Previous findings have indicated that TTP overexpression can promote apoptosis in various cells lines [46]. The results presented here using HPV-positive cervical cancer cells demonstrate the ability of TTP to inhibit cell growth. However, in this TTP-inducible system evidence of apoptosis was not observed as indication of caspase-3 activation, nuclear condensation, and DNA fragmentation was not apparent in HeLa cells expressing TTP over a 7 day time course (data not shown) indicating that TTP-mediated growth inhibition was occurring through an alternate mechanism in HeLa cells. In our findings, HeLa cells expressing TTP exhibited a flattened morphology and elevated levels of β-galactosidase activity indicating they have undergone replicative senescence. These findings are in agreement with recent results demonstrating TTP-mediated growth inhibition using a similar TTP-inducible HeLa cell model [25]. These differences in phenotypic outcome resulting from TTP expression in cells may reflect a specific variation in the ARE-containing mRNAs targeted for TTP-mediated decay in the differing cell types.

In HeLa cells, repression of viral E6 and E7 oncogene expression can trigger endogenous senescence pathways [47-50]. Although our results could be explained through the ability of TTP to inhibit E6 or E7 expression,  we did not observe any TTP-dependent changes in E6/E7 transcript level (Figure 5A). Interestingly, an AU-rich region has been identified within the 3'UTR of HPV16 E6/E7 RNA that can mediate rapid decay [51]. The results presented here (Figure 8) and that of others [36] demonstrate TTP to be abundantly expressed in normal uterine cervix. Based on these observations, it is plausible that that TTP may play a protective role in the early stages of HPV infection by targeting E6/E7 RNA for rapid degradation. However, this viral ARE is lost in cells containing integrated HPV16 genomes through the process of viral DNA linearization and host genome integration [51] and the consequences of this would make E6/E7 RNA resistant to TTP-mediated decay. This loss of post-transcriptional control, coupled with disruption of the viral E2 transcriptional repressor [52], would potentiate persistent E6/E7 oncogene expression needed for cell transformation.

Actively growing HeLa cells maintain a dormant p53 pathway and elevated telomerase activities [49]. The results presented here demonstrate the ability of TTP to promote p53 protein expression, which is consistent with senescent growth arrest that is often associated with an active p53 pathway [53]. In normal cells, p53 levels are under negative regulation of Mdm2 ubiquitin ligase and p53 pathway activation primarily involves signal-dependent escape from degradation [54,55]. Whereas in high-risk HPV-transformed cervical cancer cells, the viral oncoprotein E6 binds to p53 and with the help of the cellular ubiquitin ligase E6-AP, p53 is targeted for constitutive degradation through the ubiquitin proteasomal pathway [6,32,56].

Replicative senescence in somatic cells is in part attributed to gradual loss of telomeres, while high telomerase activity is observed in a majority of cancer cells [30]. Another characteristic of cervical cancer cell transformation is reactivation of *hTERT* gene expression, which is the catalytic component of telomerase. Although the mechanism of E6-dependent activation of *hTERT *is not entirely defined in HPV-transformed cells, current observations indicate the involvement of E6-AP in targeting a regulator of hTERT expression [5,33,57]. Furthermore, p53 can serve as a negative regulator of hTERT expression [58], suggesting that E6/E6-AP-dependent degradation of p53 may also play a causal role in *hTERT* promoter activation.

**Figure 9.**
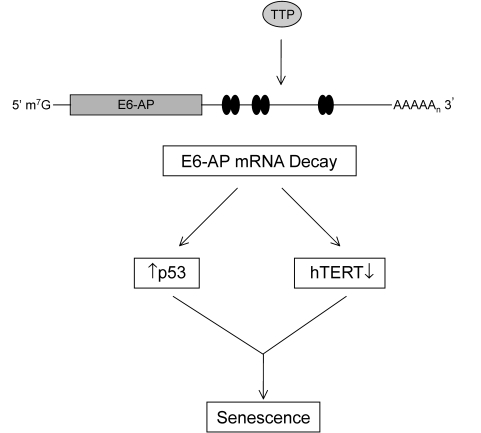
TTP-mediated regulation of E6-AP in cervical cancer cells. The binding of TTP to the ARE-containing E6-AP mRNA targets it for rapid degradation. Black ovals represent putative 3' UTR AU-rich elements (AREs). The subsequent loss of E6-AP expression allows for concurrent p53 protein stabilization and inhibition of *hTERT* transcription leading to cellular senescence.

Central to the deregulation of these factors in cervical cancer is E6-AP and the results presented here are readily explained with E6-AP being a novel target of TTP-mediated post-transcriptional regulation (Figure 9). Within the 3'UTR of E6-AP, the presence of AU-rich elements provide a binding site for TTP and this functional interaction targets E6-AP mRNA for rapid ARE-mediated decay. These results are supported by the observations that the presence of E6-AP 3'UTR to a luciferase reporter renders it susceptible to TTP-mediated downregulation and deletion of the 3'UTR from E6-AP makes it resistant to TTP-mediated mRNA decay (Figure 7). The functional consequences of TTP-mediated suppression of E6-AP leads to p53 stabilization, hTERT inhibition, and cellular senescence. These results are consistent with those using RNA interference to downregulate E6-AP expression indicating the central role E6-AP has in promoting HPV-associated cervical cancer [59].

Recent findings have demonstrated that loss of TTP expression is observed in a variety of tumor types [25,36,44,60]. Consistent with this, we also observe a similar loss of TTP in cervical cancer cells and tumors. This loss of TTP expression appears to be a critical factor in the progression of high-risk HPV-associated cervical cancer, since the presence of TTP in cervical tumor cells impedes their tumorigenic potential through rapid decay of E6-AP mRNA. Also observed with the loss of TTP expression is increased expression of the ARE-mRNA stabilization factor HuR in cervical cancers [61]. Through these combined defects of TTP loss-of-function and HuR gain-of-function, aberrant mRNA stabilization can occur leading to over-expression of cancer-associated factors in cervical cancer similar to what is seen in colon cancer [44]. Moreover, the potential of TTP to promote senescence may be through its ability to antagonize HuR-mediated stabilization of proliferative ARE-mRNAs [44,62] similar to observations showing that reduction in HuR levels in fibroblasts promoted a senescent phenotype [63].

The mechanisms underlying the loss of TTP expression in cervical cancer cells and tumors are largely undefined. The TTP gene (*ZFP36*) is located on 19q13.1 and does not appear to be a target of genomic loss or rearrangement in cervical cancer [64]. One explanation for the lack of TTP expression observed in tumor tissue may reside in epigenetic silencing of the TTP promoter. Within the proximal 3' region of the human TTP promoter lies a putative CpG island and the presence of hypermethylation of this region was observed in HeLa cells (unpublished observations). Based on this we hypothesize that epigenetic alterations occurring through changes in DNA methylation and altered chromatin structure promote TTP gene silencing in cervical tumors. This is consistent with observations demonstrating that various tumor suppressor genes have been silenced or display decreased expression resulting from abnormal promoter hypermethylation in HPV-associated cervical carcinoma [65].

The results presented here provide a novel link between post-transcriptional gene regulation and HPV-associated cervical tumorigenesis. Based on these findings we conclude that TTP promotes cellular senescence in cervical cancer cells through rapid decay of E6-AP mRNA leading to p53 protein stabilization and inhibition of *hTERT* transcription. Moreover, absence of TTP expression in cervical cancer strongly implicates that loss of TTP expression is a critical step that occurs early in HPV-mediated carcinogenesis. These findings demonstrate the novel ability of TTP to servein a tumor suppressor capacity by regulating ARE-mRNA gene expression and identify how defects in post-transcriptional regulation can contribute to tumorigenesis.

## Methods

Cell culture, DNA transfection, and adenoviral infection. Human cervical cancer cell lines HeLa (HPV18+), SiHa (HPV16+) and CaSki (HPV16+) cells were obtained from ATCC; HeLa Tet-Off cells were purchased from BD Clontech. Cells were maintained in DMEM supplemented with 10% fetal bovine serum (FBS; Hyclone); HeLa Tet-Off cell media was supplemented with 100 μg/ml G418 (Cellgro). The Tet-responsive pTRE2hyg/TTP-Flag vector was created by cloning an N-terminal Flag epitope-tagged TTP cDNA from pcDNA3-Flag-TTP (kindly provided by N. Kedersha, Brigham and Women's Hospital, Boston, MA) into pTRE2hyg (Clontech). HeLa Tet-Off cells were stably transfected with pTRE2hyg/TTP-Flag using Lipofectamine Plus (Invitrogen) according to the vendor's protocol. Stably transfected cells were selected in normal growth medium containing 100 μg/ml G418, 200 μg/ml hygromycin B (Invitrogen), and 2 μg/ml doxycycline (Dox) (Clontech) for 2-3 weeks. Individual clones were isolated by limiting dilution in 96-well plates. Positive HeLa-Tet-Off/TTP-Flag clones were screened by growing cells in the presence or absence of Dox (2 μg/ml) to induce expression of TTP-Flag, respectively; the absence of Dox allows for TTP-Flag expression. For stable cell maintenance the hygromycin B concentration was reduced to 100 μg/ml. Unless otherwise indicated, HeLa Tet-Off/TTP-Flag cells were grown in the absence of Dox for 48 hr to induce TTP-Flag expression.

HeLa Tet-Off/TTP-Flag cells were transiently transfected with p53-responsive promoter luciferase reporter vector pp53-Luc or control vector pTA-Luc (Clontech) along with control pRL-TK renilla vector (Promega) using Lipofectamine Plus. The E6-AP coding region or 3'UTR were PCR amplified from HeLa cDNA as described [66]. E6-AP coding region was cloned into the expression vector pcDNA3.1/Zeo (Invitrogen) to generate pcDNA3.1/E6-APΔ3'UTR. Luciferase reporter construct containing the E6-AP 3'UTR was prepared by cloning E6-AP 3'UTR into pcDNA3.1/Zeo containing the luciferase cDNA [66]. Cells were transfected in DMEM for 3 hr after which cells were grown in complete medium in the presence or absence of 2 μg/ml Dox for 48 hr. Transfected cells were lysed in reporter lysis buffer (Promega) and assayed for luciferase and renilla activities using the Dual-Luciferase Assay System (Promega). Luciferase reporter gene activities were normalized to renilla activity and all results represent the average of triplicate experiments.

TTP-Flag expressing adenovirus was created by cloning TTP-Flag cDNA into the shuttle vector Dual-CCM-CMV-EGFP (Vector Biolabs) that contains dual CMV promoters to drive expression of TTP-Flag and GFP. Construction of TTP-expressing adenoviral vector (AdGFP/TTP) and production of viral stocks were conducted by Vector Biolabs. Control GFP-expressing adenovirus (AdGFP) was purchased from Vector Biolabs. HeLa, SiHa and CaSki cells were infected with AdGFP or AdGFP/TTP using a MOI of 100 in serum-free DMEM for 2 hr after which FBS was added to a final concentration of 10%. 48 hr after infection, cells were harvested in SDS-PAGE lysis buffer for western blot analysis.

Immunoblot analysis. Cells were lysed in SDS-PAGE lysis buffer (50 mM Tris-HCl, pH 6.8, 100 mM DTT, 2% SDS, 0.1% bromophenol blue, 10% glycerol) and protein content was determined using a BCA protein assay with BSA as standard (Pierce Biotechnology). Where indicated, nuclear lysates were prepared by resuspending cells in lysis buffer (10 mM HEPES pH 7.9, 2 mM MgCl_2_, 10 mM KCl, 0.1 mM EDTA, 1 mM DTT, 0.5% NP-40) containing 0.5 mM PMSF and protease inhibitor cocktail (Sigma) and incubated on ice for 10 min. Cells were centrifuged at 13000 rpm for 10 min and the nuclear pellet was washed 3 times with lysis buffer. Nuclei were lysed in RIPA buffer (50 mM Tris-Cl pH 8.0, 156 mM NaCl, 4 mM EDTA, 1% SDS, 1 % Triton X-100, 1% Na-deoxycholate). Lysates (50 μg) were separated by 10% SDS-PAGE, transferred to PVDF membranes (Bio-Rad), and probed with antibodies against Flag epitope (M2; Sigma), TTP (Ab-36558, Abcam), p53 (DO-1, Calbiochem), hTERT (Ab-1, Calbiochem), and E6-AP (BD Biosciences) at dilutions specified by the vendor. Blots were stripped and then probed with antibodies against β-actin (Clone C4, MP Biomedicals) or nucleoporin (BD Biosciences). Detection and quantitation of blots were performed as described [66].

mRNA analysis. Total RNA was extracted from cells using Trizol reagent (Invitrogen). Northern blotting was performed as previously described [67] and probed with P^32^-labeled DNA probes synthesized for TTP, E6-AP and actin (Promega). cDNA synthesis and RT-PCR analysis of mRNA was accomplished as described [66]. The sequences for PCR primers used were: TTP sense, 5'-TCCACAACCCTAGCGAAGAC-3' and TTP anti-sense, 5'-GAGAAGGCAGAGGGTGACAG-3'; p53 sense, 5'-CAGCCAAGTCTGTGACTTGCACGTAC-3' and p53 antisense, 5'-CTATGTCGAAAAGTGTTT CTGTCATC-3'; hTERT sense, 5'-GTGACCGTGGTT TCTGTGTG-3' and hTERT antisense, 5'-TCGCCTGA GGAGTAGAGGAA-3'; HPV18 E6 sense, 5'-CGCGC TTTGAGGATCCAA-3' and HPV18 E6 antisense, 5'-TATGGCATGCAGCATGCG-3'; HPV18 E7 sense, 5'-TATGCATGGACCTAAGGCAAC-3' and HPV18 E7 antisense, 5'-TTACTGCTGGGATGCACACC-3'; E6-AP sense, 5'-GCTTGAGGTTGAGCCTTTTG-3' and E6-AP antisense, 5'-CCAATTTCTCCCTTCCTTCC-3'; GAPDH sense, 5'-CCACCCATGGCAAATTCCAT GGCA-3' and GAPDH antisense, 5'-TCTAGACGGCA GGTCAGGTCCACC-3'. Real-time PCR (qPCR) was performed using the 7300 Real-Time PCR Assay System (Applied Biosystems) with SYBR green PCR master mix (Applied Biosystems) and primers for E6-AP and GAPDH according to the vendor's protocol.

Cell growth and senescence. Cell growth was assayed using the MTT-based cell growth determination kit (Sigma) as previously described [68]. For cellular senescence studies, 1 x 10^4^ HeLa Tet-Off/TTP-Flag cells were grown in 35mm diameter dishes in the presence or absence of 2 μg/ml Dox. Twelve days later, the cells were stained at pH 6.0 with X-Gal (5-bromo-4-chloro-3-indolyl-β-D-galactopyranoside; Cell Signaling Technology) to visualize senescence associated-β-galactosidase (SA-β-gal) activity.

Fluorescence microscopy. HeLa Tet-Off/TTP-Flag cells were plated on coverslips in a 24-well plate and grown in the presence or absence of 2 μg/ml Dox. After 48 hrs, the cells were fixed in 4% paraformaldehyde for 10 min and permeabilized with 0.1% Triton X-100 in PBS for 5 min. The cells were blocked with 10% normal goat serum and 3% BSA diluted in PBST (PBS + 0.1% Tween-20) for 1 hr. Cells were incubated for 1 hr at RT with anti-p53 antibody (DO-1, Calbiochem; 1:100) diluted in blocking buffer. After washing, the cells were incubated with fluorescein-conjugated goat anti-mouse secondary antibody (MP Biomedicals; 1:150) for 1 hr at RT. DAPI (Invitrogen) was used for nuclear counter-staining. Coverslips were mounted on glass slides and visualized using an Axiovert 200 inverted microscope (Zeiss). Cell morphology was examined by staining fixed and permeabilized cells with DAPI and rhodamine phalloidin (Invitrogen) according to the vendor's instructions.

Telomerase activity. Telomerase activity was determined in HeLa Tet-Off/TTP-Flag lysates 48 hr after TTP induction using the PCR-based TRAP assay as previously described [69]. PCR products were resolved on a 10% non-denaturing polyacrylamide gel and visualized by silver staining [70].

Immunoprecipitations. HeLa Tet-Off/TTP-Flag (1.25 x 10^5^ cells) were grown in 100 mm diameter dishes in the presence or absence of 2 μg/ml Dox for 48 hr. Cells were lysed in polysome lysis buffer (100 mM KCl, 5mM MgCl_2_, 10mM HEPES pH 7.0, 0.5% NP-40, and 1 mM DTT) containing 100 U/ml RNase inhibitor (Ambion) and protease inhibitor cocktail (Sigma). Cytoplasmic extracts were separated from nuclei by centrifugation at 20,000g for 30 min. 700 μl of IP buffer (50 mM Tris-Cl, pH 7.4, 150 mM NaCl, 1 mM MgCl_2_, 0.05% NP-40) was added to 500 μg of lysate and immunoprecipitation of TTP-bound RNA was accomplished by incubating lysates with equal amounts (30 μg) of anti-Flag mAb or mouse IgG pre-coated to protein A/G PLUS agarose (Santa Cruz Biotechnology) overnight at 4°C. Immunoprecipitates were collected by brief centrifugation and washed 4 times with IP buffer. Total RNA was isolated using 1 ml Trizol per IP reaction and then used for cDNA synthesis [66]. Real-time PCR reactions were performed using 1 μl of cDNA. Data was plotted as 1/C_t_ to represent the abundance of E6-AP or GAPDH mRNA in each IP sample.

Immunohistochemical analysis. Immunohistochemical analysis of TTP expression was performed using cervical cancer tissue array CXC96101 (Pantomics) that contained 12 cases of normal and inflammatory tissues of cervix and 36 cases of cervical cancer graded by histology. TTP immunostaining was performed using TTP polyclonal antibody (Ab-36558, Abcam) at 8 μg/ml (1:400). Standard staining protocol was performed and stained tissue sections were evaluated for intensity of staining as described [44] using two blinded investigators (S.S and V.K.). For each tissue section, the percentage of positive cells was scored on a scale of 0 to 4 : 0 (0% positive cells), 1 (< 25%), 2 (25-50%), 3 (50-75%) or 4 (> 75%). Staining intensity was scored on a scale of 0 to 3; 0-negative, 1-weak, 2-moderate, or 3-strong. The two scores were multiplied to give an immunoreactivity score (IRS) ranging from 0 to 12, with scores in the range of 0-6 grouped in the category of Low IRS and those in the range of 7-12 representing High IRS.

Statistical analysis. The data are expressed as the mean +/- SD. Student's *t*-test was used to determine significant differences. *P*-values less than 0.05 were considered significant.
